# Conditional cash transfers and adolescent mental health in Brazil: Evidence from the 2004 Pelotas Birth Cohort

**DOI:** 10.7189/jogh.11.04066

**Published:** 2021-10-30

**Authors:** Carolina Ziebold, Cristiane Silvestre Paula, Iná S Santos, Fernando C Barros, Tiago N Munhoz, Crick Lund, David McDaid, Ricardo Araya, Annette Bauer, Emily Garman, A-La Park, Annie Zimmerman, Philipp Hessel, Mauricio Avendaño, Sara Evans-Lacko, Alicia Matijasevich

**Affiliations:** 1 Programa de Pós-graduação em Distúrbios do Desenvolvimento. Universidade Presbiteriana Mackenzie, São Paulo, Brazil; 2 Postgraduate Program in Pediatrics and Child Health, School of Medicine, Pontifical Catholic University of Rio Grande do Sul, Porto Alegre, Brazil; 3 Postgraduate Program in Epidemiology, Federal University of Pelotas, Pelotas, Brazil; 4 Postgraduate Program in Health and Behavior, Universidade Católica de Pelotas, Pelotas, Rio Grande do Sul, Brazil; 5 Faculty of Psychology, Federal University of Pelotas, Pelotas, Brazil; 6 Centre for Global Mental Health, Health Services and Population Research Department, Institute of Psychiatry, Psychology and Neuroscience, King’s College London.; 7 Alan J Flisher Centre for Public Mental Health, Department of Psychiatry and Mental Health, University of Cape Town, Cape Town, South Africa; 8 Care Policy and Evaluation Centre, Department of Health Policy, London School of Economics and Political Science, London, UK; 9 Department of Global Health & Social Medicine, King’s College London, London, UK; 10 Escuela de Gobierno Alberto Lleras Camargo, Universidad de Los Andes, Bogotá, Colombia; 11 Center for Primary Care and Public Health (Unisanté), Department of Epidemiology and Health Systems, University of Lausanne, Lausanne, Switzerland; 12 Departamento de Medicina Preventiva, Faculdade de Medicina FMUSP, Universidade de São Paulo, SP, Brazil

## Abstract

**Background:**

Young people living in poverty are at higher risk of mental disorders, but whether interventions aimed to reduce poverty have lasting effects on mental health has not been well established. We examined whether exposure to Brazil’s conditional cash transfers programme (CCT), *Bolsa Família* (BFP), during childhood reduces the risk of mental health problems in early adolescence.

**Methods:**

We used data from 2063 participants in the 2004 Pelotas Birth Cohort study. Propensity score matching (PSM) estimated the association between BFP participation at age 6 and externalising problems (Strengths and Difficulties Questionnaire – SDQ and violent behaviour) and socio-emotional competencies (Development and Well-Being Assessment questionnaire, and the Nowick-Strickland Internal-External Scale) at age 11.

**Results:**

PSM results suggest that programme participation at age of six was not significantly associated with externalising problems (*P* = 0.433), prosocial behaviour (*P* = 0.654), violent behaviour (*P* = 0.342), social aptitudes (*P* = 0.281), positive attributes (*P* = 0.439), or locus of control (*P* = 0.148) at the age of 11 years.

**Conclusions:**

Participation in BFP during childhood was not associated with improved or worsened mental health in early adolescence. While we cannot fully discard that findings may be due to adverse selection, results suggest that CCTs alone may not be sufficient to improve mental health outcomes and would be prudent to assess whether mental health interventions as an addition to CCTs may be helpful.

Poverty is a complex multidimensional concept that goes beyond lack of income, and involves health, education and living standard deprivations [1], among others. Poverty is closely linked to mental health problems during childhood and adolescence [2]. In particular, poverty during childhood and adolescence has been linked to higher risk of externalising behavioural problems, including attention-deficit/hyperactivity, oppositional defiant and conduct disorders [3]. Similarly, externalising problems experienced early in life can reduce educational and employment opportunities and increase the risk of lifetime poverty [4,5]. Mechanisms underlying this association include food insecurity [6], exposure to violence [7], diminished social support [8], dysfunctional coping strategies, and diminished social competencies [9,10]. There is also growing evidence suggesting that poverty may exacerbate caregiver’s depression which in turn may increase offspring’s mental health problems [9,11,12]. An important, yet untested hypothesis is that interventions that address poverty during childhood and adolescence may help improve the mental health of young people living in poverty [13].

Conditional cash transfers programmes (CCTs) have emerged as one of the most widely used anti-poverty policies in Low- and Middle-Income Countries (LMICs). CCTs provide cash payments on the condition that families meet specific requirements related to children´s school attendance, vaccinations or health check-ups [14]. The majority of evidence in this area has focused on evaluating CCTs impact on poverty reduction [15], child health, nutrition [16], and educational outcomes [17], finding overall positive effects of CCTs on these outcomes. Although mental health problems are not directly targeted by CCTs, some studies have examined their potential impact on the mental health of young people living in poverty [18-20]. The rationale of these studies is that because poverty may be linked to mental health problems, CCTs provide an opportunity to test whether improvements in living and social conditions associated with poverty (food security, parental supervision, access to health and education services) may exert a positive effect on mental health [13,19-21]. Prior studies have examined the effect of CCTs on depression and anxiety among youth (15 to 24 years) [22], and on behavioural problems and socio-emotional competencies, among children (<10 years) [19-21]. Whilst some find modest improvements [19-22] others show no effect [21]. To our knowledge, there has been no research on the association between CCTs, externalising problems and socio-emotional competencies among adolescents living in LMICs. Examining this issue in a LMIC context is important. On the one hand, externalising problems are more common among groups with the lowest socioeconomic status and are associated with considerable social and economic consequences over the lifetime due to, for example, higher violent offending, drug use, teenage pregnancy and school dropout [23-25]. On the other hand, it has been suggested that improved socio-emotional competencies (eg, interpersonal skills, social aptitudes and prosocial behaviour) may prevent the development of externalising behaviours [26].

Brazil’s *Bolsa Família* programme (BFP) began in 2003 and is nowadays the CCT with the largest number of beneficiaries in the world, providing financial assistance to approximately 14 million families [27]. BFP aims to both alleviate poverty by providing a cash benefit to families and reduce the inter-generational transmission of poverty by conditioning receipt of these transfers on human capital investments (school attendance, immunization, pre-natal care) [27]. Programme eligibility is based on per capita household income and household composition (pregnant/breast feeding mothers; children aged 0-17). Monthly payments are made to mothers (average US$35 per month in 2020) [27].

Most BFP`s evaluation studies have examined impacts of the programme on poverty [28,29], food security and nutrition [30,31], infant and child mortality [32,33], as well as health [34] and education services [35,36]. Only one study has examined impacts on mental health, finding a positive association of BFP with psychosocial functioning among children and adolescents (7-17 years) in an urban slum community in Northeast Brazil [34]. Evidence also suggests that an increase in BFP coverage was associated with a reduction in suicide rates, especially among women in municipalities where high coverage of the programme was maintained for 3 years or more [37].

To advance our understanding of the association between CCTs and mental health outcomes among children and adolescents, we analysed the association of BFP participation with externalising problems and socio-emotional competencies (social aptitudes, positive attributes, and locus of control). Our study is the first in analysing this relationship and unique by linking exposure to the programme relatively early in life (age 6 years) to mental health outcomes in early adolescence (age 11 years). We hypothesized that BFP at the age of six may reduce adolescent`s externalising problems and increase socio-emotional competencies at the age of 11.

## METHODS

### Study site

We used data from the 2004 Pelotas Birth Cohort study. Pelotas is a city in the south of Brazil, with an estimated population of 342 405 inhabitants in 2019. Major economic activities are commerce, services and industry [38]. In 1982, the city’s gross domestic product was 9% above the national mean, but by 2004 it was 39% lower, reflecting the relative impoverishment of the city [39]. A previous study from the 2004 Pelotas Birth Cohort found that BFP coverage – the percentage of poor people in the cohort receiving the benefit – increased from 29% in 2004 to 63% in 2010, whereas the targeting of the programme – the percentage of eligible people among the beneficiaries – remained constant at about 37% [40]. Nowadays, BFP coverage in Pelotas city is 54% [41].

### Participants

The 2004 Pelotas Birth Cohort is a population-based birth cohort of children born in Pelotas from January 1 to December 31, 2004. All live births of women residing in the urban area of the city during that year were recruited for the study (n = 4231, nonresponse rate at recruitment <1%). A detailed description of the methodology can be found elsewhere [42]. After perinatal assessment, the cohort was followed up at the ages of 3, 12, and 24 months, as well as at 4, 6 and 11 years, with follow-up rates between 87% and 96%. In the present study we included cohort participants who: 1) had complete data on sociodemographic characteristics gathered during the perinatal and 4-year assessments; 2) were living in households from the four first poorest quintiles of wealth at the age of 4; we excluded participants from the richest quintile as these participants were in principle not eligible for the BFP and only a small fraction (7.3%) reported receiving it; 3) had complete data on both BFP participation status at the age of 6 and mental health outcomes at age 11 (6.3% lost to follow-up). Study participants’ flowchart (Figure S1 in the **Online Supplementary Document**) and characteristics related with attrition are presented in Table S1 in the **Online Supplementary Document**.

### Exposure: Bolsa Família benefit at the age of 6

During follow-up when children reached the age of 6, mothers or other main caregivers were asked: “Do you receive the BFP?”. We defined BFP beneficiaries as those who responded “yes” to this question.

Eligibility: BFP eligibility is based on per capita household income and household composition, according to the information provided by families registered in the Unified Registry for Social Programmes of the Federal Government. In 2010, families with a monthly per capita income of up to R$70 (about US$42 at 2010 values) were classified as extremely poor and were eligible for the programme [27]. Families with per capita incomes between R$70.01 and R$140.00 were classified as poor and could be beneficiaries if they contained children between 0-6 years of age, or children and adolescents enrolled in school (7-17 years), or pregnant or breastfeeding woman (14-44 years) [27]. The number of families participating in BFP is also limited by quotas per municipality that are calculated on the basis of estimates of the number of families living in poverty in each municipality [43]. The entry of families into the programme follows the following order: first, families considered to be priorities (*quilombola* [ethnic-racial groups]; indigenous; recyclable material collectors; families in situations of child labour; and with members freed from slave-like labour); second, families with the lowest monthly income per capita; third, families with the highest number of children and adolescents from 0 to 17 years old [43].

Benefit: In 2010, extremely poor families received an unconditional amount of R$68 (about US$40 in 2010). Poor families received only benefits according to household composition. Both extremely poor and poor families received additional benefits dependent on the number of children in the family (0-15 years) and whether the mother was pregnant or breastfeeding (up to five additional benefits of R$22 [about US$14] per family: R$110 in 2010) [27]. An additional amount of R$33 (about US$20) was received by families with adolescents enrolled in school aged 16-17 years (up to two per family: R$66). Monthly payments are made to mothers using an electronic payment card [27].

Conditionalities: For both extremely poor and poor families, additional benefits are dependent on compliance with health and education conditionalities: 1) Children under the age of 7 are expected to comply with Brazil’s childhood immunization schedule and to make growth and development monitoring visits with the frequency recommended by the Ministry of Health [27], 2) Children between the ages of 6-17 years are expected to be enrolled in school and maintain a minimum daily school attendance of 85% (75% for ages 16-17), 3) Pregnant women have to comply with prenatal care [27], 4) Breastfeeding mothers (14-44 years) have to comply with health care controls. Schools and health centres are responsible for reporting adherence [27].

### Outcome measures

#### Externalising problems at the age of 11 years

At subsequent follow-up when children reached 11 years, mothers/main caregivers were interviewed by trained psychologists using the Strengths and Difficulties Questionnaire (SDQ) [44], an instrument designed to assess mental health problems in children and adolescents, validated in Brazil [45]. The conduct problems, hyperactivity and prosocial behaviour subscales of the SDQ were used to measure adolescent externalising problems [46]. Each subscale contains 5 items asking about the child’s behavioural problems or strengths, as for example ‘*Steals from home, school or elsewhere*’ (conduct), *‘Restless, overactive, cannot stay still for long’* (hyperactivity), or ‘*Considerate of other people's feelings’* (prosocial). Items can be answered as “Not True”, “Somewhat True” and “Certainly True”, receiving scores from 0 to 2 for each answer. The SDQ externalising score ranges from 0 to 20 and is the sum of the conduct and hyperactivity scales, where greater scores are indicative of greater externalising problems [46]. The prosocial behaviour score ranges from 0 to 10. Reversed scores were computed, where greater scores are indicative of less prosocial behaviour [46].

Violent behaviour was evaluated using a self-completed confidential questionnaire developed by the 1993 Pelotas Birth Cohort authors [47] where adolescents responded to a yes or no question: “During the last year, did you participate in a fight in which someone was injured?”.

#### Socio-emotional competencies at age 11

Three socio-emotional competencies were tested: Social aptitudes were assessed by the Social Aptitude Scale (SAS) [48], a 10-item scale designed to evaluate individual social abilities that form part of the Development and Well Being Assessment (DAWBA) [49] that has been validated in Brazil [50]. Parents were asked to rate the ability of their child, when compared with children of the same age, to read social and emotional cues rapidly in complex situations in order to guide socially skilled behaviour in ten different social situations. Total scores range between 0 and 40, where higher scores represent more social aptitudes.

Positive attributes were assessed using the Youth Strengths Inventory (YSI) version for parents, which also forms part of the DAWBA [49-51]. The first part of the YSI asks how applicable various descriptions are to the child (for example being generous, affectionate, caring), while the second part asks about positive behaviours (for example being good with friends, helpful at home, and polite). Each part has 12 items scored on a three-point Likert scale, with scores ranging from 0 to 48, where higher scores represent greater positive attributes.

Locus of Control (LoC) describes the degree to which individuals perceive that outcomes of events in their lives result from their own behaviours (internal LoC), or from forces that are external to themselves (external LoC) [52]. Adolescents’ LoC were evaluated using the 12-item version of the Nowick-Strickland Internal-External Scale (CNSIE) [52]. Each item is dichotomous, with yes-no responses, and a total score is derived by summing scores for all items. Higher scores indicate a more external LoC, while lower scores indicate greater internal LoC. The Portuguese version has shown good psychometric properties [53].

### Covariates

*Maternal characteristics at childbirth:* maternal schooling (number of completed years of formal education); maternal age; marital status (single mother or living with a partner); and number of live children, all gathered at perinatal interview.

*Child characteristics at childbirth*: sex (male and female); gestational age; weight; and skin colour (white, black, and mixed-race).

*Household wealth score at 4-year follow-up*: Household characteristics were evaluated according to the National Wealth Index questionnaire [54]. Principal component analysis was used to consolidate the following household characteristics into one measure representing the household’s wealth [55]: household head’s education; number of bedrooms; number of bathrooms (with shower and toilet); number of television sets; number of vehicles; ownership (yes/no) of assets: radio, refrigerator, DVD or video tape, freezer/duplex refrigerator; washing machine, microwave, telephone line, computer and air conditioner [55]. The wealth index allows classification of households into five wealth strata according to reference cut-off values for each municipality. Using Pelotas-specific reference values, wealth indexes were stratified as follows: 20-280 = first strata (poorest households), 281-367 = second strata, 368-475 = third strata, 476-618 = fourth strata, and 619-1478 = fifth strata (wealthiest households, who were excluded from the analysis). Even though BFP eligibility is based on per capita household income, it has been suggested [40] that the first strata of the wealth index constructed as explained above is a good proxy of BFP eligibility. It represents the poorest population, those that would be potential beneficiaries of the programme in the municipality, being less subject to temporal variability and errors of information than household income [40]. Figure S2 in the **Online Supplementary Document** shows the rates of receipt of BFP into each wealth strata.

*Maternal depressive symptoms at 6-year follow-up:* Maternal depressive symptoms during the past 7 days were assessed using the Edinburgh Postnatal Depression Scale (EPDS) [56]. The EPDS is a 10-item scale originally created for the identification of postpartum depression. Total scores range from 0 to 30, with higher scores indicating more severe depressive symptoms. Maternal depressive symptoms was defined using a cut-off of ≥13 that was validated to screen major depressive episodes among adults from the general population in Brazil [57].

### Ethical considerations

All procedures were approved by the Research Ethics Committees of the Universidade Federal de Pelotas and the Universidade de São Paulo, School of Medicine. Mothers or legal guardians of all subjects gave written informed consent. At 11 years of age, the adolescents also gave written informed assent.

### Data analysis

We first analysed the frequency and factors related to attrition between follow up at 6 and 11 years. Due to the low attrition rate (6.3%) and its lack of relationship with factors under study (Table S1 in the **Online Supplementary Document**); we performed a complete-case analysis. For main tests we adopted a level of significance of 5%.

### Treatment effects estimation

To assess the effect of BFP on adolescents’ externalising problems and socio-emotional competencies, we used propensity score matching (PSM) (Stata 16, College Station, TX) [58,59]. PSM allows mitigation of bias associated with differences in the distributions of observed covariates in BFP beneficiaries (treatment group) and non-beneficiaries (comparison group). PSM matches treatment and comparison groups according to the subject’s similarity on estimated treatment probabilities, known as propensity scores [58-60].

*Propensity score (PS)*: For this study, the PS is the probability of being a BFP beneficiary given observed covariates for the child, mother, and household. We selected BFP participation covariates using *probit* regression models and we also estimated covariates of our outcomes of interest using generalised linear models (Tables S2 and S3 in the **Online Supplementary Document**). The PS was estimated according to variables strongly associated to both BFP participation and outcomes [59]: household wealth index, maternal characteristics (age at childbirth, number of living children, maternal schooling, and maternal depressive symptoms), and child characteristics (sex and skin colour).

*Matching:* To reduce bias, we used 1:1 nearest-neighbour matching (NNM), where each beneficiary is matched with the non-beneficiary who has the closest PS, with replacement and a caliper (specification of the maximum distance at which two observations are a potential match) of 0.005 [58,59]. NNM is considered the most effective method for selecting individuals for follow-up [61]. After each model, we performed tests of balance to check that each covariate did not significantly differ between the treated and comparison groups (non-significant mean differences) [58-60]. In addition, we present density-distribution plots of propensity scores for BFP and non-BFP participants. These plots allow us to visually check the overlap or common support condition which ensures that respondents with the same values on the PS have a positive probability of being both BFP participants and nonparticipants.

*Average treatment effect on the treated:* PSM allows estimation of the average treatment effect on the treated (ATT), where the treatment effect is computed by taking the average of the difference between the outcome of BFP beneficiaries and the outcome of eligible non-beneficiaries [58,60].

*Sensitivity analysis:* We tested two alternative matching methods: kernel matching (Epanechnikov type), where all BFP beneficiaries were matched with a weighted average of all non-beneficiaries with weights that were inversely proportional to the distance between the PS of beneficiaries and non-beneficiaries, and radius matching (with caliper of 0.005) which uses as many comparison cases (not only the nearest-neighbour) as are available within the caliper [62]. We also performed subgroup analyses to test whether the results of the PSM would vary among participants in the first (poorest) and fourth (richest) wealth strata. Finally, we also present the results of simple regression models testing the association of BFP and adolescent mental health adjusting by the same covariates included in the PSM.

## RESULTS

### Sample differences before and after propensity score matching (PSM)

The total sample included 2063 participants, where 873 (42.3%) were BFP beneficiaries at the age of 6 years and 1190 (57.7%) were non-beneficiaries. **Table 1** illustrates sample characteristics. 20.4% of beneficiaries fell within the first wealth strata (poorest), with most concentrated in the second (28.1%) and third (33.1%) wealth strata, whereas 18.4% were in the fourth wealthiest strata. In terms of BFP coverage, 61.2% of cohort participants from the first wealth strata were beneficiaries (Figure S2 in the **Online Supplementary Document**). Before PSM, beneficiaries presented higher mean scores on SDQ externalising problems (β = 0.14, SE = 0.04, *P* < 0.001) and external locus of control (β = 0.06, SE = 0.01, *P* < 0.001) compared with non-beneficiaries.

**Table 1:** Sample description: Sociodemographic characteristics and means of externalising problems and socio-emotional competences, Pelotas 2004 Birth Cohort. (N = 2063)

Child and household characteristics	Total		BFP		Non-BFP	
**N or Mean**	**% or SD**	**N or Mean**	**% or SD**	**N or Mean**	**% or SD**
Child’s sex:						
Female	1004	48.7	446	51.1	558	46.9
Weight at birth (grammes)	3163.0	538.7	3167.9	525.1	3159.4	548.6
Skin colour:						
White	1336	64.8	505	57.9	831	69.8
Black	281	13.6	140	16.0	141	11.9
Mixed	446	21.6	228	26.1	218	18.3
Maternal schooling (years)	7.15	2.97	6.04	2.67	7.96	2.91
Maternal age at childbirth	25.45	6.92	26.12	6.96	24.96	6.84
Mother’s number of children at childbirth	1.07	1.33	1.58	1.53	0.70	1.01
Mother living with partner at childbirth	1700	82.4	729	83.5	971	81.6
Wealth Score (4 years)	405.75	117.45	368.41	111.33	433.00	114.31
Wealth Strata (4 years):						
1^st^-Poorest	291	14.1	178	20.4	113	9.5
2^nd^	436	21.1	245	28.1	191	16.1
3^rd^	702	33.0	289	33.1	413	34.7
4^th^	634	30.7	161	18.4	473	39.6
Maternal depressive symptoms (6 years)	387	18.8	216	24.7	171	14.4
Child’s mental health (11 years):						
SDQ externalising problems*	4.9	4.4	5.3	4.7	4.6	4.1
SDQ less prosocial behaviour†	0.8	1.4	0.8	1.4	0.8	1.4
Violent behaviour‡	274	13.7	124	14.6	150	13.0
Social aptitudes	20.6	3.3	20.5	3.3	20.7	3.3
Positive attributes	38.2	6.9	38.2	7.2	38.3	6.7
Locus of control	6.6	1.9	6.6	2.0	6.2	1.9
Total	2063	100.0	873	42.3	1190	57.7

BFP – *Bolsa Família* Programme, N – number of observations, SD – standard deviation, SDQ – Strengths and Difficulties Questionnaire

*Conduct plus hyperactivity items. Higher scores indicate greater externalising problems.

†Prosocial subscale is reverse scored. Higher scores indicate less prosocial behaviour.

‡Any 12-month physical aggression that caused injuries, N = 2007 adolescent report.

**Table 2** presents the *probit* model with the covariates predicting BFP participation. Beneficiaries were more likely than non-beneficiaries to be non-white, to reside in a household with lower wealth, to have mothers with higher odds of presenting depressive symptoms, lower education, lower age at childbirth, and with more children. We did not find differences according to child sex (β = 0.04, SE = 0.06, *P* = 0.474), weight at birth (β = 0.000003, SE = 0.0001, *P* = 0.961), and maternal marital status (β = -0.001, SE = 0.08, *P* = 0.986) between the two groups. However, as child sex was significantly associated with our outcomes of interest (Tables S2-S3 in the **Online Supplementary Document**) we included this variable in the PSM.

**Table 2:** *Probit* regression model of participation in the BFP

Child and household characteristics	Coef.	SE	*P-*value	95% CI
Maternal age at childbirth	-0.01	0.01	0.011	-0.02 - 0.00
Mother’s number of children	0.29	0.03	<0.001	0.23 - 0.35
Maternal schooling years	-0.09	0.01	<0.001	-0.11 - -0.07
Child’s skin colour:				
White (Reference)	1			
Black	0.06	0.09	0.470	-0.11 - 0.24
Mixed	0.20	0.07	0.007	0.06 - 0.34
Poorest wealth strata	0.27	0.09	0.002	0.10 - 0.44
Child’s sex:				
Male (Reference)	1			
Female	0.06	0.06	0.310	-0.06 - 0.18
Maternal depression (yes)	0.22	0.08	0.004	0.07 - 0.37
Test statistics: *χ*^2^ = 371.99, *P* < 0.001, PseudoR^2^ = 0.13				

BFP – *Bolsa Família* Programme, Coef. – coefficient in the *Probit* regression model, SE – standard deviation, CI – confidence interval, PseudoR^2^ – pseudo-R squared statistics

**Table 3** shows covariates balance before and after PSM using the NNM technique. After PSM the mean differences between groups were in all cases non-significant, meaning that PSM succeeded in removing differences in the covariates' distributions. Figure S3 in the **Online Supplementary Document** shows the overlap of the PS between the matched beneficiaries and non-beneficiaries, meaning that, according to the observed covariates, each matched pair had equal probability of being eligible for BFP.

**Table 3:** Covariates balance before and after nearest-neighbour propensity score matching

Child and household characteristics		BFP, mean	Non-BFP, mean	T-test, *P*- value
Maternal age at childbirth	Before	26.12	24.96	<0.001
	After	25.94	25.49	0.191
Mother’s number of children	Before	1.58	0.70	<0.001
	After	1.48	1.43	0.482
Mother schooling years	Before	6.04	8.00	<0.001
	After	6.11	5.97	0.281
Black skin colour	Before	0.16	0.12	0.006
	After	0.16	0.15	0.640
Mixed skin colour	Before	0.26	0.18	<0.001
	After	0.26	0.23	0.158
First wealth strata	Before	0.20	0.10	<0.001
	After	0.19	0.22	0.230
Female sex	Before	0.51	0.47	0.060
	After	0.51	0.52	0.528
Maternal depression	Before	0.25	0.14	<0.001
	After	0.24	0.22	0.205

BFP – *Bolsa Família* Programme

**Table 4** presents the number of cases included (on support) and excluded (off support) in the PSM and the percentage of bias reduced after PSM. The matched sample included more than 96% of beneficiaries and all non-beneficiaries. Bias reduction ranged between 63.3% to 80.9%.

**Table 4:** Nearest-neighbour propensity score matching: common support and bias reduction*

	Matched BFP			Matched Non-BFP			Bias	Bias	Bias reduction
	**On support**		**Off support**	**On support**		**Off support**	**Before matching**	**After matching**	
	**N**		**N**	**N**		**N**	**Mean**	**Mean**	**%**
**Externalising problems:**									
SDQ-Externalising problems†	849	24		1190	0		31.2	5.1	76.1
SDQ-Less prosocial‡	849	24		1190	0		31.2	5.1	76.1
Violent behaviour§	819	33		1155	0		31.3	4.4	80.9
**Socio-emotional competencies:**									
Social aptitudes	847	21		1184	0		31.1	6.1	63.3
Positive attributes	850	22		1190	0		31.2	4.0	77.2
Locus of control‖	806	21		1132	0		31.1	5.9	72.5

BFP – *Bolsa Família* Programme, N – number of observations, SDQ – Strengths and Difficulties Questionnaire

*Matched by sex, ethnicity, maternal schooling, household wealth score, maternal age at childbirth, maternal depressive symptoms, and number of live children at birth.

†Conduct plus hyperactivity items.

‡Prosocial subscale is reverse scored.

§Any 12-month physical aggression that caused injuries, N = 2007 adolescent report.

‖N = 1959 adolescent report.

### BFP participation, externalising problems and socio-emotional competencies

Using the nearest-neighbour matched sample we calculated the ATT of BFP participation at age 6 on externalising problems and socio-emotional competencies at age of 11. **Table 5** shows that BFP was not significantly associated with SDQ externalising problems at age of 11 (*P* = 0.433), or with prosocial (*P* = 0.654) or violent (*P* = 0.342) behaviour.

**Table 5:** Effect of BFP participation at 6 years on mental health at 11 years among 2004 Pelotas Birth Cohort participants*

	Average treatment effect on the treated		
**Outcome**	**BFP vs Non-BFP difference**	**SE**	***P*-value**
**Externalising problems:**			
SDQ-Externalising problems†	-0.05	0.31	0.433
SDQ-Not Prosocial‡	-0.04	0.09	0.654
Violent behaviour§	0.01	0.02	0.342
**Socio-emotional competencies:**			
Social aptitudes	-0.15	0.25	0.281
Positive attributes	-0.07	0.48	0.439
Locus of control‖	0.19	0.13	0.148

BFP – *Bolsa Família* Programme, SDQ – Strengths and Difficulties Questionnaire, SE – standard error

*Treatment effects estimator: Nearest-neighbor Propensity score matching. Matched by sex, ethnicity, maternal schooling, household wealth score, maternal age at childbirth, maternal depressive symptoms, and number of live children at birth.

†Conduct plus hyperactivity items. Higher scores indicate greater externalising problems.

‡Prosocial subscale is reverse scored. Higher scores indicate less prosocial behaviour.

§Any 12-month physical aggression that caused injuries, N = 2007 adolescent report.

‖N = 1959 adolescent report.

As shown in **Table 5**, we did not find any significant association of BFP with socio-emotional competencies – social aptitudes (*P* = 0.281), positive attributes (*P* = 0.439), or locus of control (*P* = 0.148) – at the age of 11.

### Sensitivity analysis

Tables S5-S7 and Figures S4-S5 in the **Online Supplementary Document** show that kernel and radius matching methods had similar results as those presented with the NNM in terms of ATT (non-significant association between BFP and all outcomes assessed) and overlap assumption, even showing higher bias reduction. Because NNM is considered the most effective method for selecting individuals for follow-up [61], we decided to present its results in the main analysis nonetheless. Stratified analysis also showed a non-significant association between BFP and all outcomes assessed among those in the poorest and wealthiest strata (Tables S8-S9 in the **Online Supplementary Document**). Finally, simple regression models also showed non-significant associations between BFP and adolescent mental health after adjusting for the covariates included in the PSM (Table S10 in the **Online Supplementary Document**).

## DISCUSSION

We analysed the longitudinal association between BFP at age 6 and externalising problems and socio-emotional competencies at age 11 among a population-based birth cohort of young people in Brazil. We found that BFP participation at the age of 6 was not associated with externalising problems, prosocial and violent behaviour or socio-emotional competencies in early adolescence (age 11 years).

Our findings differ from the positive effect of BFP on psychosocial functioning that Shei et al [34] found among young people in Northeast Brazil. It could be argued that their cross-sectional study design, measures (the SF-10 Health Status questionnaire), data analysis (propensity score weighting) and population (children and adolescents (7-17 years) living in an urban slum in Salvador de Bahía) significantly differed from our study. Further studies are needed to verify if the impact of BFP on mental health outcomes varies according to the time frame (short-term vs longer-term), type of outcome (externalising, and internalising disorders or positive well-being) and the age group. It is particularly important to replicate these studies in different regions of the country to understand if cultural and socioeconomic backgrounds, quality of services and implementation features of the programme, can exert different impacts of BFP on mental health in young people.

Our results also differ from those found by Ozer et al [20] who examined the cross-sectional effect of the Mexico’s Oportunidades CCT on behaviour problems among children (4-6 years) living in poor rural areas. They found a 10% decrement in aggressive and oppositional symptoms (assessed by the Behaviour Problems Index – BPI). Although, like our study, they used propensity score matching to define comparison groups, our longitudinal design, measures, and population differ considerably from theirs. In Nicaragua, Macours et al [21] performed a randomised controlled trial (RCT) to evaluate the effect of the Atención a Crisis CCT on behavioural problems (assessed by the BPI) and social competencies (assessed by the Denver Developmental Screening Test) among children (3-7 years) nine months after the programme began and two years after its end. They found a significant positive effect of the programme on social competencies but, as in our study, they found non-significant effect on behavioural problems.

In spite of crude associations showing that beneficiaries would have higher levels of externalising problems and external locus of control, our results using PSM suggest that BFP is not associated with worsened or improved externalising mental health problems and socio-emotional competencies among low-income adolescents living in Pelotas. Our interpretation of this null association between BFP and adolescent mental health is consistent with the fact that young people living in poverty are exposed to risk factors for externalising problems, as community and domestic violence [7], diminished social support [8], and caregiver’s depression [9,12], those may not be properly addressed by the programme. On the other hand, BFP includes no specific mental health interventions, such as preventive interventions aiming to develop socio-emotional competencies, or screening and referral for mothers and children presenting with mental disorders. It is also possible that the pressures of conditionalities for the BFP may bring stress to families as observed in another CCT [63], reducing the potential positive benefit that poverty reduction may have on mental health.

A secondary finding of this study was the association between BFP receipt and maternal depressive symptoms. As in other studies [11,12,64], we also found that these depressive symptoms were related with child behavioural problems. These results highlight the need for further research to evaluate potential complementary mental health interventions that can be delivered among families participating in the programme. For instance, an RCT in Liberia [65] compared the effect of CTs vs CTs plus Cognitive Behaviour Therapy (CBT) on antisocial behaviour among vulnerable young males. The combined arm of cash plus CBT showed a dramatic reduction in antisocial behaviour one year later, while CTs on their own had no effect.

As stated earlier, mental health problems are not only more likely among people living in poverty [2], they can also reduce the life-chances and increase the risk of lifetime poverty [4,5]. In consequence, it would be prudent to assess whether mental health interventions may be included as part of poverty reduction strategies [66].

### Strengths and limitations

The present study contributes to understanding the association between CCTs and early adolescent mental health in LMICs, using individual longitudinal data. We analysed data from a large population-based birth cohort with low attrition rates, using validated instruments and exploiting longitudinal assessments. In addition, we used PSM to address potential selection and confounding. However, our findings need to be interpreted with caution, and a number of important limitations need to be considered. First, the interpretation of our treatment effect estimates from PSM analyses relies on the assumption that there are no unmeasured confounding variables producing biased effect estimates. For instance, many factors correlated with poor mental health may also be associated with participation into BFP, biasing our estimates against positive mental health effects. Second, our study was limited to evaluate outcomes at only one follow-up and future cohort waves are needed to evaluate the stability of the association between BFP participation and mental health during youth. Third, we did not have data for many variables that could allow for an improved understanding of the BFP effects as amount of benefit, compliance with conditionalities, use and quality of health, educational and social services, and years of receipt of BFP. Nevertheless, we were able to verify (Table S11 in the **Online Supplementary Document**) that the results did not change when we excluded from the analyses 149 controls (13%) those became beneficiaries at age of 11. We also found that ‘losing’ (46%) vs ‘maintaining’ (54%) the benefit at the age of 11 was not associated with outcomes assessed (Table S12 in the **Online Supplementary Document****)**.

#### Implications for research and policy

The results of the current study reinforce the need to further evaluate mental health outcomes of CCT in LMICs [13,67], and to identify which aspects of the programmes (eg, conditionalities, amount of money, length of benefit, and complementary health, social and educational interventions) can help to prevent or reduce the severity of mental health problems among young people. Our study also raises the need for replication in other Brazilian regions and to systematically use mixed-methods evaluations to verify if the BFP is improving mental health outcomes in young people.

Given that households living in poverty are exposed to preventable risks to mental health, greater attention needs to be paid to the mental health of programme beneficiaries. Our research provides an opportunity to advise policymakers to also consider incorporating mental health interventions into their poverty reduction strategies. Furthermore, evaluation of evidence-based complementary tailored interventions that can be delivered to prevent mental health problems among BFP beneficiaries, specifically mothers, children and adolescents, would be recommended [65].

## Additional material


Online Supplementary Document
