# Supplementary material for: Conditional cash transfers and adolescent mental health in Brazil: Evidence from the 2004 Pelotas Birth Cohort
Source: J Glob Health. 2021 Oct 30;11:04066. doi: 10.7189/jogh.11.04066 (PMC8564883; doi:10.7189/jogh.11.04066)
Supplement: Online Supplementary Document [file jogh-11-04066-s001.pdf]

Figure S1 Flow chart of 2004 Pelotas Birth Cohort participants included in the study

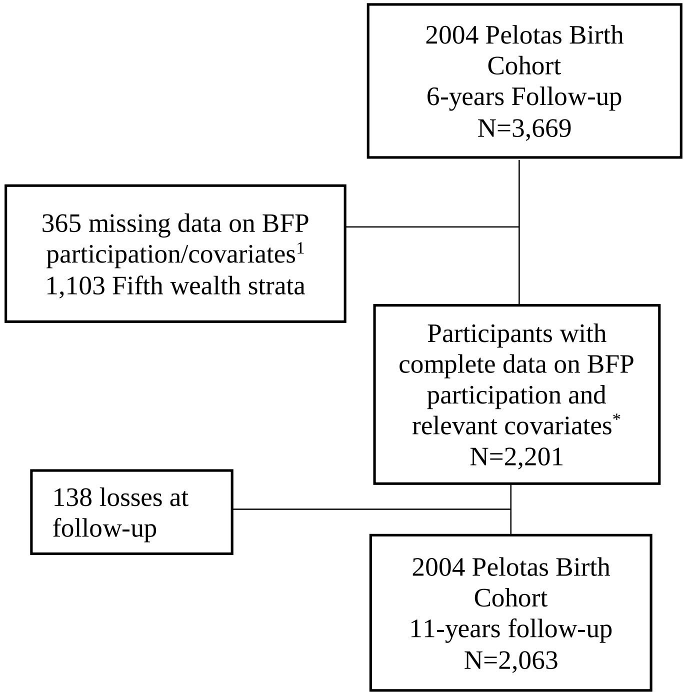

*Missing information in variables gathered during the perinatal assessment and at the 4 and 6-years follows-up

Table S1. Child/adolescent and household predictors of attrition at 11-years follow-up, 2004 Pelotas Birth Cohort (N=2,201)

Child and household predictors			Total	Attrition	No-attrition	OR 95% CI	P^*
**BFP**	**Beneficiary**	N	949	76	873	1.41	0.077
		%	43.1	8.0	92.0	0.96-2.07	
**Sex**	**Female**	N	1,058	54	1,004	**0.66**	**0.021**
		%	48.1	5.1	94.9	**0.46-0.94**	
**Weight at birth (gr.)**		M	3,158.91	3,097.44	3,163.02	1.00	0.214
		SD	536.54	501.16	538.69	1.00-1.00	
**Child's skin colour**							
	**White**	N	1,424	88	1,336	Reference	
		%	64.7	6.2	93.8		
	**Black**	N	302	21	281	0.88	0.638
		%	13.7	7.0	93.1	0.53-1.48	
	**Mixed**	N	475	29	446	0.82	0.365
		%	21.6	6.1	93.9	0.52-1.27	
**Household wealth score**		M	403.31	367.17	405.75	1.00	**0.030**
		SD	117.79	117.36	117.45	1.00-1.00	
**Maternal schooling (years)**		M	7.11	6.49	7.15	0.98	0.545
		SD	2.96	2.69	2.97	0.91-1.05	
**Maternal age at childbirth**		M	25.38	24.30	25.45	0.97	**0.044**
		SD	6.88	6.31	6.92	0.94-1.00	
**Mother's number of children**		M	1.08	1.20	1.07	1.09	0.307
		SD	1.34	1.50	1.33	0.93-1.28	
**Mother living with partner**		N	1,810	110	1,700	0.90	0.657
		%	82.2	6.1	93.9	0.58-1.41	
**Maternal depressive symptoms**		N	422	35	387	1.22	0.353
		%	19.2	8.3	91.7	0.80-1.84	
**Total**		N	2,201	138	2,063	-	-
		%	100.0	6.3	93.7		

BFP – *Bolsa Família* Programme, SD – standard deviation, OR – Odds Ratio, 95% CI – 95% confidence interval, gr. – grams.

* Results of logistic Regression models predicting the odds of attrition at follow-up versus no attrition.

Figure S2 Percentage of participants receiving BFP in each wealth strata

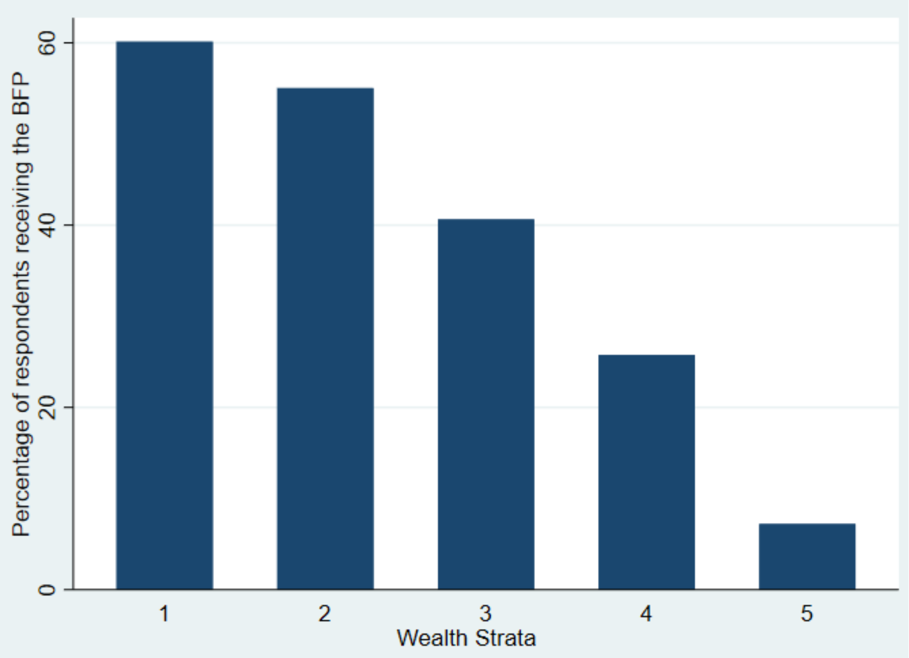

BFP – *Bolsa Família* Programme

Table S2. Bivariate analysis: predictors of externalising problems at age of 11 years, 2004 Pelotas Birth Cohort N=2,063.

		SDQ Externalising		SDQ less prosocial		Violent Behaviour	
		β	*P*	β	*P*	OR	*P*
**Female sex**		**-0.24**	**<0.001**	**-0.14**	**0.023**	**0.37**	**<0.001**
**Weight at birth**		**-0.0001**	**0.029**	-0.00001	0.663	1.00	0.454
**Maternal age at childbirth**		**-0.01**	**<0.001**	-0.01	0.051	0.99	0.266
**Mother living with partner**		**-0.14**	**0.003**	-0.14	0.074	**0.61**	**0.001**
**Number of live children**		0.01	0.717	-0.02	0.466	0.97	0.414
**Maternal schooling (years)**		**-0.04**	**<0.001**	-0.02	0.126	1.00	0.888
**Gestational age at birth**		-0.01	0.316	-0.01	0.520	0.99	0.798
**Child skin colour**	**White**		Reference		Reference		Reference
	**Black**	**0.12**	**0.041**	0.17	0.057	1.37	0.084
	**Mixed**	**0.13**	**0.006**	0.08	0.331	1.02	0.901
**Household wealth score**		**-0.001**	**<0.001**	**-0.001**	**0.002**	1.00	0.806
**EPDS score 6 years**		**0.04**	**<0.001**	**0.03**	**<0.001**	1.01	0.267
**Bolsa Família 6 years**		**0.14**	**0.001**	-0.003	0.961	1.14	0.311

EPDS – Edinburgh postpartum depression scale, – SDQ – Strengths and Difficulties Questionnaire, β – beta coefficient in the regression model, OR – Odds Ratio.
Generalised linear models (logistic models for violent behaviour) estimates.

Table S3 Bivariate analysis: predictors of socio-emotional competencies at age of 11 years. Pelotas Birth Cohort N=2,063.

		Social aptitudes		Positive attributes		Locus of control	
		β	*P*	β	*P*	β	*P*
**Female sex**		**0.03**	**<0.001**	**0 .06**	**<0.001**	**0.18**	**0.035**
**Weight at birth**		**0.00001**	**0.039**	-0.0001	0.403	-0.0001	0.526
**Maternal age at childbirth**		**0.001**	**0.012**	**0.001**	**0.025**	-0.01	0.187
**Mother living with partner**		0.004	0.680	0.04	**0.001**	-0.16	0.152
**Number of live children**		-0.001	0.581	0.003	0.315	**0.14**	**<0.001**
**Maternal schooling (years)**		**0.003**	**0.022**	0.002	0.089	**-0.09**	**<0.001**
**Gestational age at birth**		0.003	0.057	0.003	0.077	0.02	0.239
**Child skin colour**	**White**	Reference		Reference		Reference	
	**Black**	-0.02	0.128	-0.01	0.487	**0.31**	**0.016**
	**Mixed**	-0.0001	0.983	-0.002	0.823	**0.29**	**0.007**
**Household wealth score**		**0.0001**	**<0.001**	**0.0001**	**0.004**	**-0.02**	**<0.001**
**EPDS score 6 years**		**-0.002**	**0.001**	**-0.01**	**<0.001**	**0.02**	**0.005**
**Bolsa Família 6 years**		-0.01	0.181	-0.003	0.740	**0.36**	**<0.001**

EPDS – Edinburgh postpartum depression scale, β – beta coefficient in the generalised linear models.

Figure S3. Overlap assumption: Distribution of the propensity score before and after matching

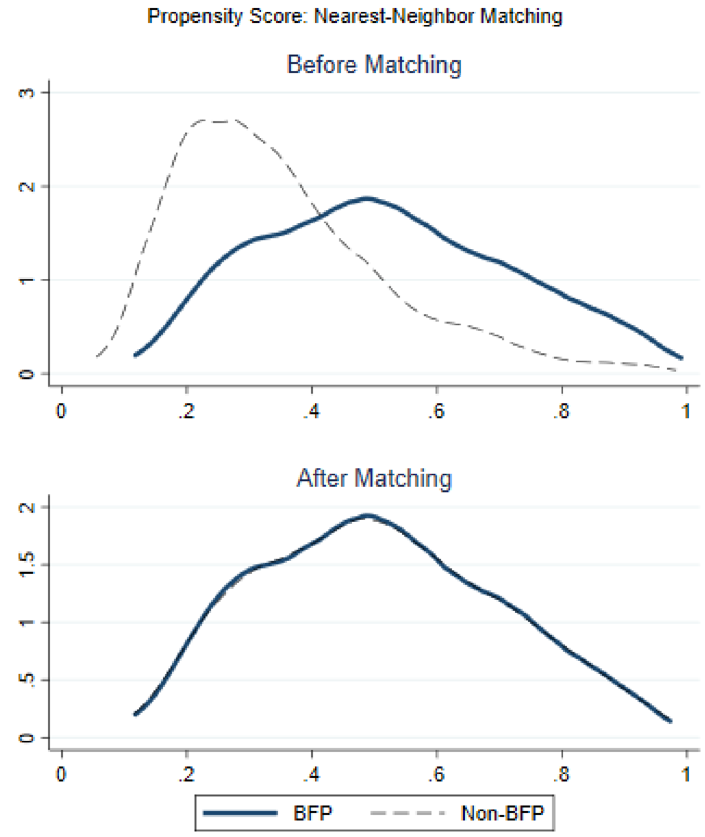

BFP – *Bolsa Família* Programme

Table S4. Kernel matching: Association between BFP participation at 6 years and mental health at 11 years among 2004 Pelotas Birth Cohort participants

**Outcome**	**Average Treatment effect on the Treated BFP vs Non-BFP**		
	**Difference**	**SE**	***P***
**Externalising problems**			
SDQ-Externalising problems	0.26	0.23	0.137
SDQ-Not Prosocial	-0.01	0.07	0.449
Violent behaviour	0.02	0.02	0.415
**Socio-emotional competencies**			
Social aptitudes	0.06	0.18	0.373
Positive attributes	-0.01	0.37	0.493
Locus of control	0.12	0.11	0.273

BFP – *Bolsa Família* Programme, SE – standard error, SDQ – Strengths and Difficulties Questionnaire.

Table S5. Kernel propensity score matching: common support and bias reduction

	Matched BFP		Matched Non-BFP		Bias	Bias	Bias
	On support	Off support	On support	Off support	Before matching	After matching	Reduction
	N	N	N	N	Mean	Mean	%
**Externalising problems**							
SDQ-Externalising problems	870	3	1.190	0	31.2	3.5	87.3
SDQ-Less Prosocial	870	3	1.190	0	31.2	3.5	87.3
Violent behaviour	849	3	1.155	0	31.3	3.5	87.5
**Socio-emotional competencies**							
Social aptitudes	865	3	1.184	0	31.1	3.5	86.7
Positive attributes	869	3	1.190	0	31.2	3.5	87.2
Locus of control	825	2	1.132	0	31.1	3.6	86.8

BFP – *Bolsa Família* Programme, SDQ – Strengths and Difficulties Questionnaire.

Figure S4. Kernel propensity score matching: Distribution of the propensity score before and after matching

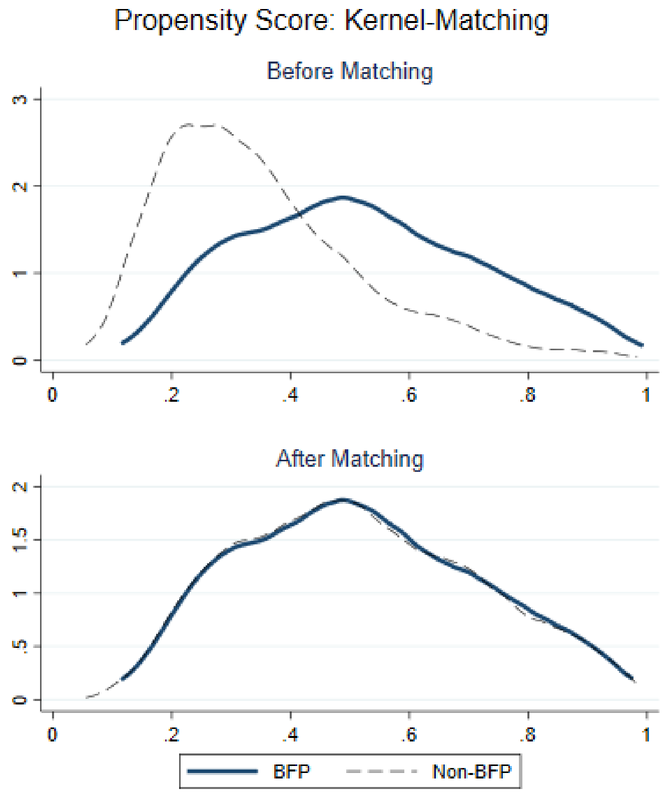

BFP – *Bolsa Família* Programme

Table S6. Radius matching: Association between BFP participation at 6 years and mental health at 11 years among 2004 Pelotas Birth Cohort participants

Outcome	Average Treatment effect on the Treated BFP vs Non-BFP		
	**Difference**	**SE**	***P***
**Externalising problems**			
SDQ-Externalising problems	0.14	0.25	0.286
SDQ-Not Prosocial	-0.04	0.08	0.302
Violent behaviour	0.02	0.02	0.190
**Socio-emotional competencies**			
Social aptitudes	0.06	0.19	0.763
Positive attributes	0.28	0.39	0.243
Locus of control	0.10	0.11	0.356

BFP – *Bolsa Família* Programme, SDQ – Strengths and Difficulties Questionnaire, SE – standard error.

Table S7. Radius propensity score matching: common support and bias reduction

	Matched BFP		Matched Non-BFP		Bias	Bias	Bias
	On support	Off support	On support	Off support	Before matching	After matching	Reduction
	N	N	N	N	Mean	Mean	%
**Externalising problems**							
SDQ-Externalising problems	849	24	1.190	0	31.2	3.6	87.9
SDQ-Less Prosocial	849	24	1.190	0	31.2	3.6	87.9
Violent behaviour	819	33	1.155	0	31.3	3.3	87.5
**Socio-emotional competencies**							
Social aptitudes	847	21	1.184	0	31.1	3.2	88.4
Positive attributes	850	22	1.190	0	31.2	3.0	88.3
Locus of control	806	21	1.132	0	31.1	4.8	80.2

BFP – *Bolsa Família* Programme, SDQ – Strengths and Difficulties Questionnaire.

Figure S5. Radius propensity score matching: Distribution of the propensity score before and after matching

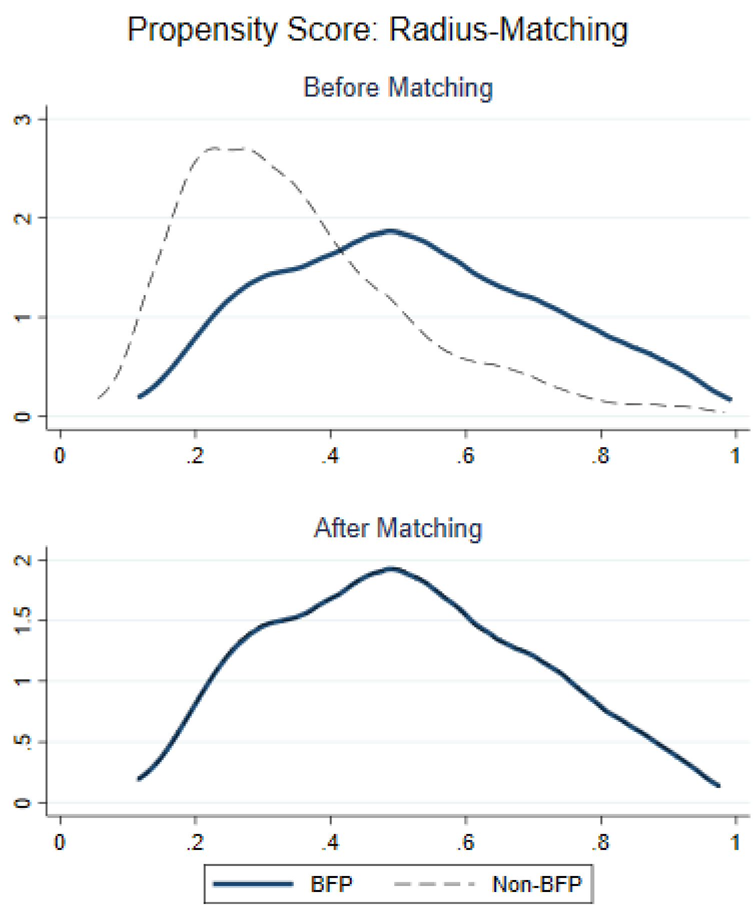

BFP – *Bolsa Família* Programme

Table S8. Association between BFP participation at 6 years and mental health at 11 years among 2004 Pelotas Birth Cohort participants in the first wealth strata* (N=291).

Outcome	Average Treatment effect on the Treated		
	BFP vs Non-BFP		
	Difference	SE	P
**Externalising problems**			
SDQ-Externalising problems	-0.04	0.80	0.482
SDQ-Less Prosocial	0.10	0.26	0.343
Violent behaviour	0.01	0.06	0.459
**Socio-emotional competencies**			
Social aptitudes	0.18	0.52	0.366
Positive attributes	-0.59	1.16	0.306
Locus of control	0.02	0.33	0.477

BFP – *Bolsa Família* Programme, SDQ – Strengths and Difficulties Questionnaire, SE – standard error.
* Treatment effects estimator: Nearest-neighbor Propensity score matching. Matched by sex, ethnicity, maternal schooling, maternal age at childbirth, maternal depressive symptoms, and number of live children at birth.

Table S9. Association between BFP participation at 6 years and mental health at 11 years among 2004 Pelotas Birth Cohort participants in the fourth wealth strata[*] (N=634).

Outcome	Average Treatment effect on the Treated BFP vs Non-BFP		
	**Difference**	**SE**	***P***
**Externalising problems**			
SDQ-Externalising problems	0.13	0.57	0.410
SDQ-Less Prosocial	-0.08	0.14	0.278
Violent behaviour	0.07	0.05	0.084
**Socio-emotional competencies**			
Social aptitudes	-0.56	0.52	0.140
Positive attributes	0.04	0.85	0.482
Locus of control	0.28	0.28	0.159

BFP – *Bolsa Família* Programme, SDQ – Strengths and Difficulties Questionnaire, SE – standard error.
[*] Treatment effects estimator: Nearest-neighbor Propensity score matching. Matched by sex, ethnicity, maternal schooling, maternal age at childbirth, maternal depressive symptoms, and number of live children at birth.

Table S10. Generalised linear models: association between BFP and externalising problems and socio-emotional problems[*]

Outcomes	Bivariate models			Multivariate models†		
	β	SE	*P*	β	SE	*P*
**Externalising problems**						
SDQ-Externalising problems	**0.14**	**0.04**	**<0.001**	0.06	0.04	0.204
SDQ-Less Prosocial	0.01	0.08	0.914	-0.06	0.09	0.511
Violent behaviour	0.19	0.12	0.120	0.20	0.15	0.174
**Socio-emotional competencies**						
Social aptitudes	-0.01	0.01	0.283	-0.002	0.01	0.794
Positive attributes	-0.004	0.01	0.647	0.003	0.01	0.747
Locus of control	**0.06**	**0.01**	**<0.001**	0.02	0.02	0.288

BFP – *Bolsa Família* Programme, SDQ – Strengths and Difficulties Questionnaire, SE – standard error.
[*] Outcomes were included in the models as scalar dependents variables with Gamma distribution using the log-link function, except for violent behaviour (logit models)
† Adjusting by sex, ethnicity, maternal schooling, household wealth strata, maternal age at childbirth, maternal depressive symptoms, and number of live children at birth.

Table S11. Association between BFP participation at 6 years and mental health at 11 years excluding those who became beneficiaries at age of 11[*] (N=1,914).

Outcome	Average Treatment effect on the Treated<br>Losing vs Maintaining BFP		
	**Difference**	**SE**	***P***
**Externalising problems**			
SDQ-Externalising problems	0.40	0.31	0.096
SDQ-Less Prosocial	0.04	0.10	0.354
Violent behaviour	-0.02	0.03	0.192
**Socio-emotional competencies**			
Social aptitudes	0.07	0.24	0.386
Positive attributes	-0.13	0.50	0.397
Locus of control	0.12	0.14	0.210

BFP – *Bolsa Família* Programme, SDQ – Strengths and Difficulties Questionnaire, SE – standard error.

[*]We removed those who became beneficiaries at age of 11 (N=149, 13% of the non-beneficiaries at 6 years). In these models, the control group includes only those who were not beneficiaries at both 6- and 11-years follow-ups. Treatment effects estimator: Nearest-neighbor Propensity score matching. Matched by sex, ethnicity, maternal schooling, household wealth strata, maternal age at childbirth, maternal depressive symptoms, and number of live children at birth.

Table S12. Association between changing BFP status and mental health at 11-years follow-up (2004 Pelotas Birth Cohort, N=872)*

Outcome	Average Treatment effect on the Treated<br>Losing vs Maintaining BFP		
	Difference	SE	*P*
**Externalising problems**			
SDQ-Externalising problems	-0.40	0.44	0.182
SDQ-Less Prosocial	-0.03	0.14	0.406
Violent behaviour	-0.05	0.03	0.080
**Socio-emotional competencies**			
Social aptitudes	0.34	0.29	0.239
Positive attributes	-0.39	0.68	0.283
Locus of control	-0.18	0.19	0.178

BFP – *Bolsa Família* Programme, SDQ – Strengths and Difficulties Questionnaire, SE – standard error.

[1]Effect of 'losing' the benefit (N=401, 46.0%) versus 'maintaining' the benefit (N=471, 54.0%). Treatment effects estimator: Nearest-neighbor Propensity score matching. Matched by sex, ethnicity, maternal schooling, household wealth strata, maternal age at childbirth, maternal depressive symptoms, and number of live children at birth.
